# Development and physicochemical evaluation of a gelatine-based hydrogel tube for potential use in veterinary glaucoma implants

**DOI:** 10.2478/jvetres-2026-0015

**Published:** 2026-03-19

**Authors:** Piotr Szatkowski, Martyna Fröhlich, Oliwia Grałek, Magdalena Tabor, Edyta Molik, Zuzanna Flis

**Affiliations:** Faculty of Materials Science and Ceramics, AGH University of Krakow, 30-059 Kraków, Poland; Department of Animal Biotechnology, Faculty of Animal Science, University of Agriculture in Krakow, 31-120 Kraków, Poland

**Keywords:** companion animals, gelatine stent, glaucoma, hydrogel, implant

## Abstract

**Introduction:**

Glaucoma is an eye disease in dogs and cats that increases intraocular pressure. There is no treatment method that is effective, safe and economical; however, scaffold drainage systems and hydrogels offer new therapeutic possibilities. The aim of this research was to develop a hydrogel implant for ophthalmic applications.

**Material and Methods:**

Solutions at 5%, 10% and 15% concentrations of food-grade porcine gelatine and 15% and 16% concentrations of chemically purified gelatine with 19, 20, 21 mL or 25 mL of 2.5% glutaraldehyde as a crosslinking agent per 10 mL of distilled water were candidate formulations for the hydrogel. Visual and tactile assessments were made to choose the best candidate. The implant was a thin tube with an aluminium core and a Teflon foil mould. Curing was by immersion in ethanol. Ionic conductivity and compressive strength tests were performed on cylinder-shaped samples of the hydrogel. The best candidate hydrogel was tested calorimetrically for water content and its microstructure was examined for fluid transport capacity.

**Results:**

The analyses confirmed the beneficial effect of alcohol on the structure. Hydrogels cured in ethanol were stiffer and more homogeneous and stable than those which were not, and the latter were eliminated. A hydrogel made with 1.8 g of 15% gelatine concentration, 19 mL of glutaraldehyde and 10 mL of distilled water was characterised by the highest resistance to standard compressive force. The highest ionic conductivity was obtained for the same sample. Thermal analysis showed that hydrogels stored in water contained significantly more weakly bound water (~0.53 g), whereas ethanol-conditioned hydrogels contained substantially less (~0.14 g), indicating a reduced water uptake and altered hydration profile due to alcohol conditioning. A hydrogel material based on gelatine crosslinked with glutaraldehyde was developed, from which a thin, small-diameter tube implant was made.

**Conclusion:**

These studies have shown that the implant could potentially provide a solution for minimally invasive glaucoma treatment in small animals, but the testing phase must be continued.

## Introduction

Glaucoma is a group of eye diseases characterised by a chronic increase in intraocular pressure (IOP), leading to damage to the retina and optic nerve and, as a consequence, to irreversible blindness in domestic animals – mainly dogs and cats (17). In veterinary medicine, glaucoma is divided into primary, which is often genetically determined, and secondary, which results from other eye diseases such as cataracts, uveitis or lens luxation. In the initial stage, the disease is often asymptomatic, which delays diagnosis and effective treatment. In predisposed breeds, primary glaucoma occurs more often and develops faster. Predisposed dog breeds include the Afghan hound, Akita Inu, Alaskan Malamute, cocker spaniel, beagle, basset, Chihuahua, chow chow, Dalmatian, fox terrier, golden retriever, Hungarian vizsla, Newfoundland, Norwegian elkhound, Pembroke Welsh corgi, poodle, Rhodesian ridgeback, setter, shar pei, Siberian husky, Shiba Inu and whippet (11, 15). Predisposed cat breeds include the Burmese (10, 18). Primary glaucoma is more common and progresses more rapidly. Glaucoma appears to be less common in cats than in dogs, but this difference may reflect underdiagnosis of glaucoma in cats due to the insidious onset and relatively subtle clinical signs in cats, which include dilated pupils and enlarged eyes (21).

Because of the large number of mainly canine predisposed breeds, the treatment of glaucoma is a major veterinary problem, as confirmed by epidemiological and clinical data. In purebred dogs glaucoma is found in about 1%, which is the highest rate among animal species (8). Epidemiological data from 1964–2002 show an increase in the incidence of primary glaucoma: from 0.29% to 0.89% (8). The primary goal of treatment is to reduce IOP as quickly as possible to preserve the function of the optic nerve and the comfort of the animal (17, 25). Therapy begins with pharmacological treatment: eye drops or oral agents counteracting the production of aqueous humour or supporting its outflow (17). The primary advantage of a topical preparation, *e.g*. eye drops, is the reduced incidence of adverse systemic effects. However, the strong protective barrier of the eye forces topical ophthalmic preparations to be highly concentrated, which in some cases may cause adverse systemic effects, especially in smaller animals. Orally administered agents, mainly carbonic anhydrase inhibitors, are commonly associated with adverse effects in both humans and animals (24). If pharmacotherapy proves ineffective, surgical treatment becomes necessary (25).

In recent years, there has been a dynamic development of neuroprotective, genetic and cellular approaches that may change the paradigm of glaucoma treatment. One such strategy is the use of new IOP-lowering drugs, such as latanoprostene bunod. In a study conducted on a beagle with hereditary open-angle glaucoma, latanoprostene bunod caused a decrease in intraocular pressure and pupil constriction (5). In recent years, significant breakthroughs have been made in the treatment of glaucoma in animals thanks to the development of gene and cell therapies. Innovative methods allow for simultaneous reduction of IOP and protection of retinal ganglion cells and the optic nerve (13). Another innovative glaucoma treatment strategy is the use of mesenchymal stem cells, which show promising neuroprotective effects by secreting growth factors and cytokines (20). Of particular note are new surgical techniques and the use of implants in veterinary ophthalmology. Those techniques have been shown to effectively lower IOP in dogs, often outperforming traditional methods such as cyclophotocoagulation (3, 6). Future perspectives in glaucoma therapy with implants include the exploration of scaffolds and hydrogels as drug carriers and regenerative support structures (20). Hydrogels and scaffolds may also become part of surgical procedures, offering safe, long-term and less traumatic solutions in veterinary glaucoma treatment.

One of the key aspects of medical technology development, including that of innovative glaucoma treatment methods, is the use of biocompatible materials such as hydrogels. These find wide application because of their flexibility, mechanical stability and structural modification possibilities. The currently used definition describes hydrogels as two-or multi-component systems consisting of a three-dimensional network of polymer chains and water filling the space between macromolecules. Hydrogels are most often classified according to their origin as natural or synthetic. Synthetic polymeric hydrogels combine acceptable biocompatibility with appropriately matched mechanical and degradation properties. They can be synthesised using many methods with a wide range of modifications. The most commonly used synthetic hydrogels are 2-hydroxyethyl methacrylate, polyvinyl alcohol, polyethylene glycol and polyacrylates (19, 22). Natural hydrogels can be divided into three groups: protein-based materials, polysaccharide-based materials and those derived from cell-free tissues. Those with the widest application are usually built from proteins and extracellular matrix components, thereby supporting many cellular functions, being biocompatible, bioactive and exploitable in biomedical applications. The XEN Gel Stent, commonly referred to as the XEN implant, is a sub-conjunctival, minimally invasive surgical device created to treat primary open-angle glaucoma. The stent is a small tube made of porcine gelatine crosslinked with glutaraldehyde, with good stability and biocompatibility with the eye’s physiological environment. It is an elastic and durable collagen implant introduced *ab interno*, meaning through a minimally invasive corneal incision from the front of the eye. Its task is to drain aqueous humour from the anterior chamber to the sub-conjunctival space through the scleral canal, leading to a reduction in intraocular pressure. Unlike other minimally invasive glaucoma surgical procedures, it does not require a gonioscopic view during surgery (1, 7). The aim of this study was to develop a hydrogel implant for ophthalmic applications translating the XEN Gel Stent’s material selection from human medicine to veterinary. The method proposed and studied in this report may represent a promising direction in the field of glaucoma treatment, expanding the spectrum of veterinary treatment methods.

## Material and Methods

The materials used to create the hydrogel implant structure were food-grade porcine type A gelatine powder with 180 bloom and 20 mesh, and chemically purified analytical-grade type A gelatine powder with high purity and maximum heavy metal content of 0.001% (POCH, part of Avantor Performance Materials, Gliwice, Poland). Pure 2.5% glutaraldehyde was the hydrogel crosslinking agent (WARCHEM, Zakręt, Poland), and 99.8% anhydrous ethyl alcohol was put to use to modify surface layers and create an environment that additionally formed an antifungal and antibacterial barrier (POCH).

**Fig. 1.**
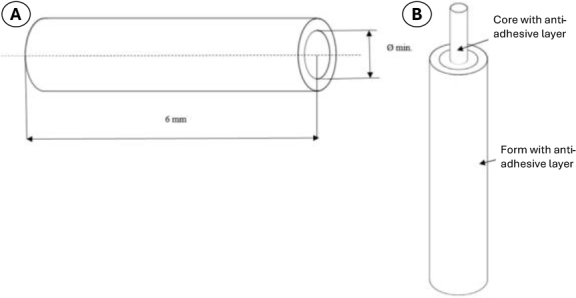
Schematic of the hydrogel implant designed for veterinary glaucoma surgical use

**Hydrogel material manufacturing method.** The requirements for the designed material in use in the physiological eye environment were a high elasticity range, easy formability, stability in an aqueous environment, appropriate mechanical compression strength and ionic conductivity. The manufacturing technology for the tube-shaped implant needed to permit minimisation of diameter, guarantee dimensional repeatability and quality, and cost comparatively little. The tubiform implant designed is shown in Fig. 1.

A 3-mm-diameter aluminium rod coated with Teflon provided the core in the developed hydrogel implant manufacturing technology. Its anti-adhesive properties did not interfere with the hydrogel implant dimensions and allowed easy removal. The shape-stabilising form for the tube was made of Teflon foil and had a diameter of 5 mm. The Teflon foil and Teflon-coated aluminium rod constituted the mould for casting the crosslinking solution of chemically purified gelatine and glutaraldehyde. After the material was sufficiently gelled, indicated by a colour change to orange, the external Teflon foil tube was removed. The manufactured tube with the core still inside was placed in 99.8% ethyl alcohol for an hour to increase strength, and then the core was smoothly removed to avoid altering the dimensions of the obtained hydrogel tube.

**Development of hydrogel material formulation.** Research on the developed hydrogel began by investigating the gelatine’s response to glutaraldehyde and determining its conditions. For this purpose, 5, 10 and 15% food-grade gelatine solutions were prepared. The appropriate masses of food-grade gelatine were weighed on an analytical balance. and combined with distilled water. Specifications of the solutions are shown in Table 1.

**Table 1.** Food-grade gelatine solutions and formulations

Concentration (%)	Gelatine mass (g)	Distilled water volume (mL)
5	0.526	10
10	1.120	10
15	1.765	10

Ten-millilitre volumes of distilled water were measured into a beaker using a measuring cylinder. Food-grade gelatine was dissolved in the water at high temperature while the temperature was measured constantly using a laser thermometer to note the point of complete dissolution. The solution was continuously stirred using a magnetic stirrer. Then, after the complete dissolution of the food-grade gelatine, 20 mL of 2.5% glutaraldehyde was added, and the changes occurring during crosslinking were observed and their relative speeds across the range of hydrogels were noted. Approximately 1 min after introducing the aldehyde into the solution, a colour change of the gelatine solution to orange was observed.

The hydrogels made with 5%, 10% and 15% concentrations of food-grade gelatine were evaluated for the effectiveness of crosslinking and for shape retention. Subsequently, the hydrogel made with the concentration of food-grade gelatine solution which was observed to be optimal was compared with one made of chemically purified gelatine. As before, 20 mL of 2.5% glutaraldehyde was added to each sample after complete dissolution of gelatine in water (the temperature at which this occurred also being measured as previously), and the mixture was stirred on a magnetic stirrer until a gel was formed. The crosslinking changes were observed and their relative speeds in both hydrogels was noted.

These hydrogels were placed in water to investigate their behaviour in an aqueous environment. An additional hydrogel sample made of 15% chemically purified gelatine solution likewise made with 1.765 g per 10 mL of distilled water and crosslinked with 20 mL of 2.5% glutaraldehyde was prepared and placed in 99.8% ethyl alcohol. The samples were left for 72 h and then subjected to shape and preliminary strength analysis.

To examine the influence of the amount of glutaraldehyde on the resulting shape and viscosity of the hydrogel, additional samples were prepared using 15% solutions of food-grade and chemically purified gelatine, with the amount of glutaraldehyde added changed to 25 mL. The samples were then placed in an aqueous environment and left for 7 days. After this time, observations of the crosslinking and shape retention of the samples were carried out.

The next stage involved the refinement of the recipe for the designed hydrogel material. The gelatine which exhibited better properties, manifested in a stiffer structure and shape, was the material used exclusively from this stage. Chemically purified gelatine was the selection. A marginally higher concentration of gelatine and a marginally larger aldehyde volume were evaluated for their effect on the hydrogels’ properties. Hydrogel samples in the form of small cylinders were prepared according to the method described above with formulation variations as presented in Table 2. Subsequently, all samples were placed in glass jars in a 99.8% ethyl alcohol environment and left for 7 d.

**Table 2.** Small-increment formulation changes to hydrogel samples

Gelatine type	Concentration (%)	Sample No.	Glutaraldehyde addition (mL)	Storage environment
	15	1	19	
Chemically purified	15	2	21	Ethanol 99.8%
	16	3	20	
	15	4	20	

**Fig. 2.**
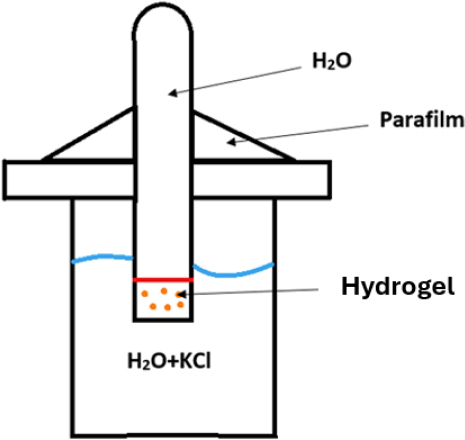
Schematic diagram of the setup for measuring the ionic conductivity of hydrogels

With these optimal parameter ranges identified, the four hydrogel formulations were comprehensively characterised to establish their performance profiles as implant materials. They were evaluated as four samples immersed in water without ethanol immersion and as four samples immersed in water and subsequently in ethanol for 72 h. A static compression test to appraise IOP resistance was conducted using a RetroLine tensile testing machine from ZwickRoell (Ulm, Germany). The measurement parameters were selected and controlled using testXpert software (ZwickRoell). The measurement parameters were uniaxial compression with a preload of 0.3 MPa and test speed of 1 mm/min. To investigate the ionic conductivity of the hydrogels and model their fluid transfer capacity, a setup as shown in Fig. 2 was designed. A plastic tube was filled with water and sealed with a hollow stopper containing the hydrogel under investigation. The tube was immersed by placing it in the perforated cap of a plastic container filled with a 9% potassium chloride solution and sealed with parafilm. An Elmetron CC-411 conductometer and an Elmetron EC-60 0925/04 electrode were used to measure conductivity (Zabrze, Poland).

Differential scanning calorimetry (DSC) analysis was conducted to investigate the water content of the formulation with the best compression resistance and ionic conductivity. This step and the subsequent ones were also carried out on one sample immersed only in water (which served as a reference) and one sample immersed in water and cured in ethanol. Measurements in a Mettler-Toledo DSC analyser (Greifensee, Switzerland) were carried out in the temperature range of 5–250°C. The heating rate was 10 K/min. Micrographs were taken of the microstructure of the hydrogel samples to illustrate their pore size and distribution and facilitate fluid transfer capacity prediction. A Keyence VHX-5000 digital microscope captured the images with a 3D scanning attachment with a resolution of 1 μm (Osaka, Japan). Profiles were also obtained using the microscope’s surface roughness measurement capability to evaluate surface characteristics affecting bacterial adhesion and implant biocompatibility. Additionally, using 3D modelling in the microscope software, an analysis of the size distribution and arrangement of pores in the cross-section of the samples was performed at a magnification of 100×. Based on the 3D model, the size and distribution of pores in the cross-section of the ethanol-cured sample and the uncured reference sample were compared.

## Results

**Formulation optimisation.** The crosslinking and shape retention evaluation led to the 5% food-grade gelatine solution hydrogel being rejected for both deficiencies, and the 10% hydrogel was also rejected because of shape instability (Table 3). The 15% gelatine solution yielded a hydrogel with a stable shape and was therefore used for further research.

**Table 3.** Comparison of the quality of food-grade gelatine hydrogels by gelatine concentration

Concentration (%)	Dissolution temperature (°C)	Crosslinking	Hydrogel shape
5	55.0	Partial	No shape
10	55.2	Complete	Impermanent shape
15	58.0	Complete	Preserved shape

The 15% food-grade gelatine hydrogel and the 15% counterpart made from chemically purified gelatine differed in crosslinking speed and dissolution temperature. These properties of these hydrogels are presented in Table 4.

**Table 4.** Comparison of the properties of food-grade and chemically purified gelatine hydrogels

Gelatine type	Concentration (%)	Dissolution temperature (°C)	Crosslinking speed
Food-grade	15	58.0	Average
Chemically purified	15	43.2	High

The hydrogels also had dissimilar properties of structural integrity correlated with gelatine type and immersion environment. The results obtained demonstrated significantly better shape retention by chemically purified gelatine. Additionally, the hydrogel immersed in ethanol exhibited a noticeable improvement in stiffness and structural uniformity. Table 5 shows the advantages of this hydrogel. The reversibility of this behaviour was verified by placing the sample previously immersed in ethanol back into water and leaving it for a period of time.

**Table 5.** Comparison of the properties of food-grade and chemically purified gelatine hydrogels after 72 h immersion

Gelatine type	Concentration (%)	Immersion environment	Shape retention
Chemically purified	15	Ethanol	Preserved shape, durable, stiff and stable
Chemically purified	15	Water	Preserved shape, numerous cracks on the surface
Food-grade	15	Water	Partial disintegration

It was possible to recognise the best crosslinking agent volume for the formulation through observations of the effect of an increase in the volume of glutaraldehyde to 25 mL. After a 7-d immersion in water, both the hydrogel made of chemically purified gelatine and the one made of food-grade gelatine exhibited weaker crosslinking compared to the gels crosslinked by 20 mL of glutaraldehyde. The sample made of chemically purified gelatine presented a stiffer, yet still watery structure and slight shape retention, while the sample made of food-grade gelatine did not retain its shape and disintegrated upon touch. The elimination of the 25-mL variants of both types of gelatine ensued from this result. The hydrogel sample made of chemically purified gelatine and crosslinked with 20 mL remained unchanged and retained its original shape and structure when fixed in ethanol and then placed back in an aqueous environment.

**Fig. 3.**
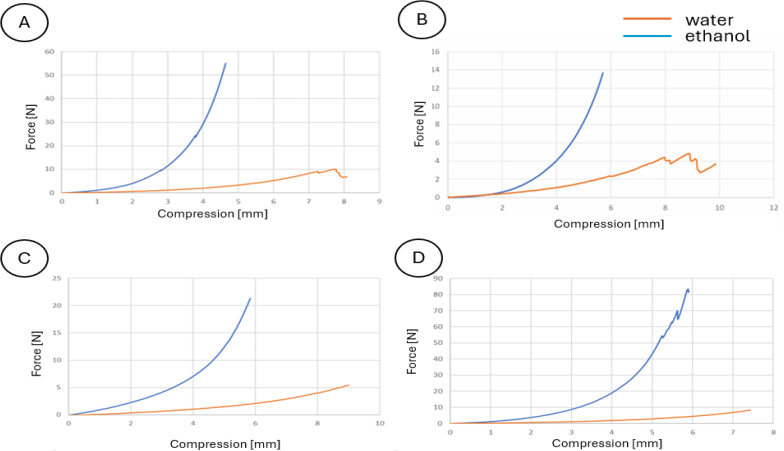
Effect of immersion environment on the compressive strength of all types of hydrogel samples. A – sample No. 1 with a glutaraldehyde concentration of 15% and the addition of 19 mL of glutaraldehyde; B – sample No. 2 with a glutaraldehyde concentration of 15% and the addition of 21 mL of glutaraldehyde; C – sample No. 3 with a glutaraldehyde concentration of 16% and the addition of 20 mL of glutaraldehyde; D – sample No. 4 with a concentration of 15% and the addition of 20 mL of glutaraldehyde. Blue line – sample immersed in ethanol; orange line – sample immersed in water

**Implant material performance evaluation.** The results of compressive strength measurements for the four types of hydrogel samples prepared based on the recipes in Table 2 and immersed in an aqueous environment and in a 99.8% ethyl alcohol environment for 72 h are presented in Fig. 3. The data are relative lengths of shortening under a standard force for each sample.

**Fig. 4.**
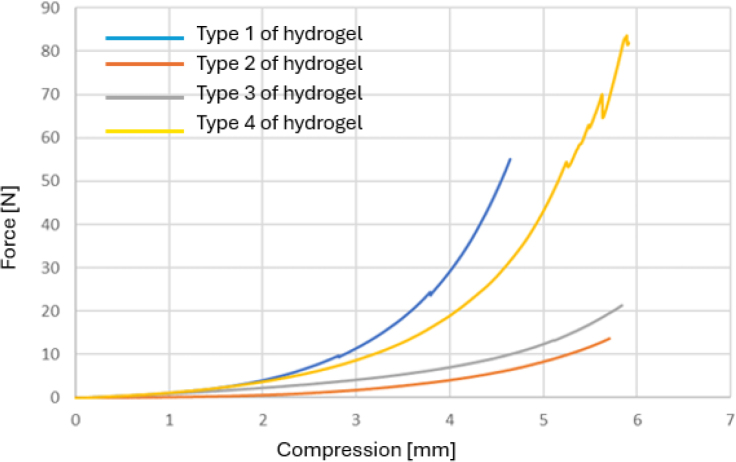
Comparison of compressive strength for different hydrogel formulations immersed in 99.8% ethanol. Type 1 – hydrogel of 15% gelatine concentration and 19 mL glutaraldehyde; Type 2 – hydrogel of 15% gelatine concentration and 21 mL glutaraldehyde; Type 3 – hydrogel of 16% gelatine concentration and 19 mL glutaraldehyde; Type 4 – hydrogel of 15% gelatine concentration and 20 mL glutaraldehyde

For each of the tested samples, the same standard force compressed hydrogels immersed in 99.8% ethanol less than those immersed only in water. The formulation selection for the hydrogel with the highest compressive strength was made based on a comparison only of the compressive strength of the four samples immersed in ethanol for 72 h. The smallest relative shortening at the same force value was exhibited by sample No. 1 (Table 6). Figure 4 shows a comparison of the transfer of compressive stresses for the tested hydrogels.

**Table 6.** Results of the compression test of hydrogels immersed in alcohol

	Relative shortening under given force (mm)				
Sample No.	10 N	20 N	30 N	40 N	50 N
1	3.04	3.65	4.06	4.26	4.55
2	5.24	-	-	-	-
3	4.67	5.82	-	-	-
4	3.24	4.13	4.55	4.97	5.23

1 No. 1 – hydrogel of 15% gelatine concentration and 19 mL glutaraldehyde; No. 2 – hydrogel of 15% gelatine concentration and 21 mL glutaraldehyde; No. 3 – hydrogel of 16% gelatine concentration and 19 mL glutaraldehyde; No. 4 – hydrogel of 15% gelatine concentration and 20 mL glutaraldehyde

**Fig. 5.**
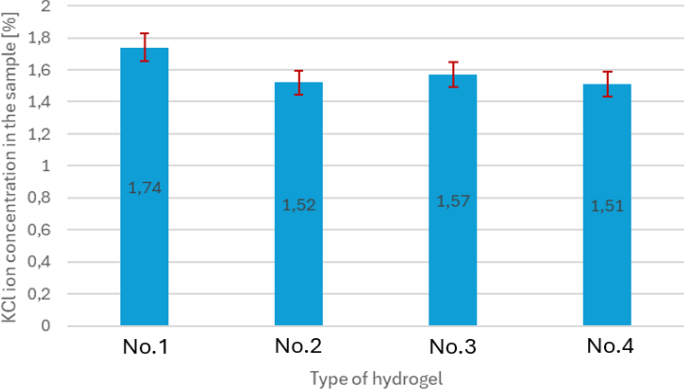
Comparison of KCl ion concentrations in the test tubes (where the solution before passing through the hydrogel is the ion source in the system) for the tested samples. No. 1 – hydrogel of 15% gelatine concentration and 19 mL glutaraldehyde; No. 2 – hydrogel of 15% gelatine concentration and 21 mL glutaraldehyde; No. 3 – hydrogel of 16% gelatine concentration and 19 mL glutaraldehyde; No. 4 – hydrogel of 15% gelatine concentration and 20 mL glutaraldehyde

**Fig. 6.**
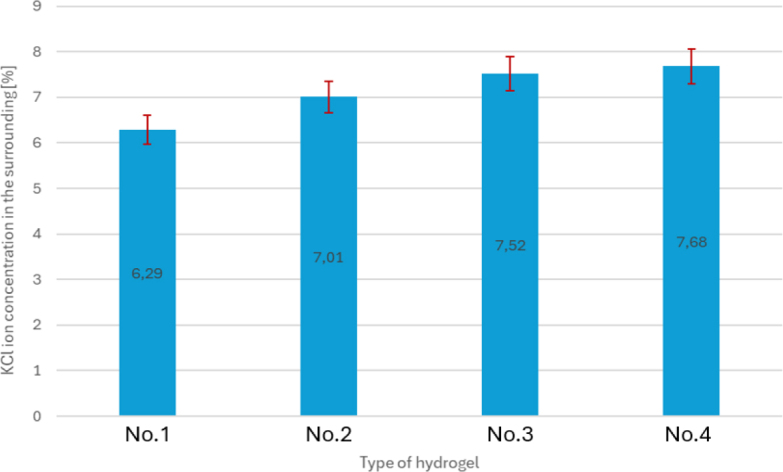
Comparison of KCl ion concentrations in the containers (after passing through the hydrogel) for the tested samples. No. 1 – hydrogel of 15% gelatine concentration and 19 mL glutaraldehyde; No. 2 – hydrogel of 15% gelatine concentration and 21 mL glutaraldehyde; No. 3 – hydrogel of 16% gelatine concentration and 19 mL glutaraldehyde; No. 4 – hydrogel of 15% gelatine concentration and 20 mL glutaraldehyde

Figures 5 and 6 show the interdependence of KCl ion concentrations in the test tubes and those in the containers. The higher the concentration of KCl ions in the test tube, the more efficiently (the faster) a given hydrogel allowed KCl ions to pass from the container to it. The highest concentration of KCl ions in the test tube along with the lowest concentration of KCl ions in the container was obtained for sample No. 1

**Fig. 7.**
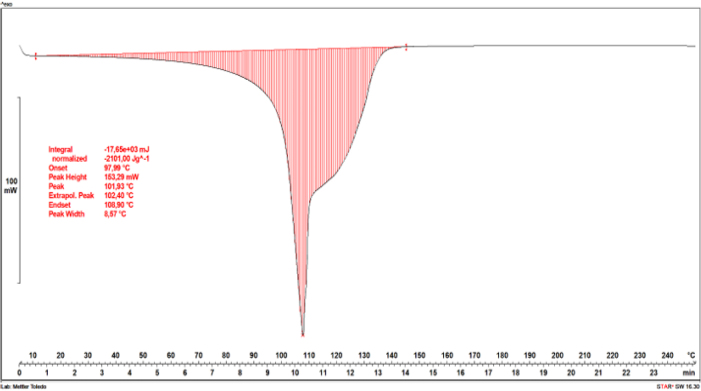
Differential scanning calorimetry curve of a hydrogel sample immersed in water but not alcohol cured, with a marked transformation region

**Fig. 8.**
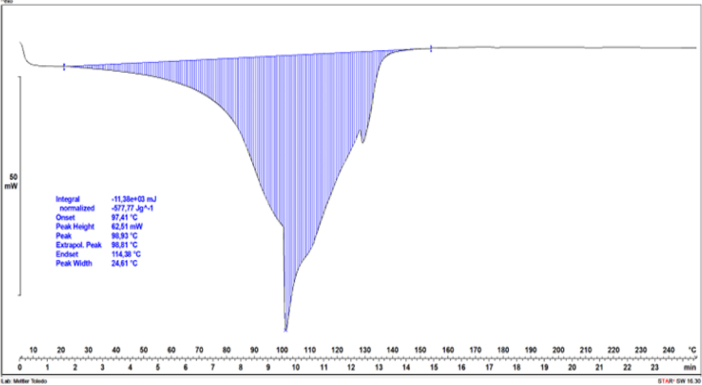
Differential scanning calorimetry curve of a hydrogel sample cured in 99.8% ethanol, with a marked transformation region

The dependencies of the heat flow rate on temperature in the studied range are exhibited for hydrogel formulation No. 1 sample immersed in water (Fig. 7) and an identical hydrogel sample immersed in 99.8% ethyl alcohol (Fig. 8).

For both tested samples, characteristic thermal transformations associated with heat absorption for water evaporation contained in the hydrogels were observed. For the hydrogel sample immersed in water (Fig. 7), two endothermic transformations were distinguished.

The dependencies of the heat flow rate on temperature in the studied range are exhibited for hydrogel formulation No. 1 sample immersed in water (Fig. 7) and an identical hydrogel sample immersed in 99.8% ethyl alcohol (Fig. 8). For both tested samples, characteristic thermal transformations associated with heat absorption for water evaporation contained in the hydrogels were observed. For the hydrogel sample immersed in water (Fig. 7), two endothermic transformations were distinguished. The first, at around 110°C, resulted from the evaporation of water weakly bound in the hydrogel structure, while the second occurred in the range of 115–145°C and was accompanied by the evaporation of the water most strongly bound in the structure. In the hydrogel sample cured in ethanol (Fig. 8), there were more thermal transformations associated with the evaporation of water with varying degrees of binding in the material structure. The most-weakly bound water was evaporated in two transformations at temperatures around 100°C. At a temperature of about 115°C, water more integrally built into the hydrogel network was removed from the hydrogel. The last noticeable endothermic transformation took place at about 135°C and was associated with the removal of the water most strongly bound in the hydrogel structure. For both hydrogel samples, there were no further thermal transformations in the range of 145–250°C, which meant that the material had thermal stability in this range. The value of the water evaporation energy for the hydrogel sample immersed in water amounted to 2,101 J/g (Fig. 7). In the case of the sample immersed in ethyl alcohol, this value was almost four times lower at 577.77 J/g (Fig. 8).

At the temperature of evaporation of the most-weakly bound water, the energy measured for the hydrogel sample immersed in water was 1,197.60 J/g, while for the sample immersed in alcohol it was 318.86 J/g. Assuming the heat of vaporisation of water at 100°C to be 2,257 J/g, it was mathematically estimated that the mass of the most weakly bound water in the uncured hydrogel sample was 0.53 g, while in the cured sample it was 0.14 g. This means that the amount of water in the hydrogel sample immersed in water was significantly greater than that in the hydrogel sample immersed in alcohol. The difference in the masses of water in the samples is related to the effect of ethyl alcohol on the hydrogel structure.

Using a digital microscope, a comparison was made of the influence of alcohol immersion on the microstructure of the hydrogel with the selected formulation. A sample of the hydrogel was analysed after immersion for 72 h in a 99.8% ethyl alcohol environment. A reference sample was made as the hydrogel with the same formulation immersed for 72 h in water only. The software used allowed a detailed analysis of the hydrogel surface at different magnifications. At 100×, visible pores were observed in both samples. The water-only sample’s pores were smaller in diameter and unevenly distributed throughout the sample cross-section. In contrast, the alcohol-cured sample’s pores were locally accumulated and significantly larger in diameter. The hydrogel sample immersed in water was more transparent than the hydrogel immersed in alcohol, the indication of which were the visible differences in the hydrogel colour shades resulting from different degrees of refraction of incident light.

**Fig. 9.**
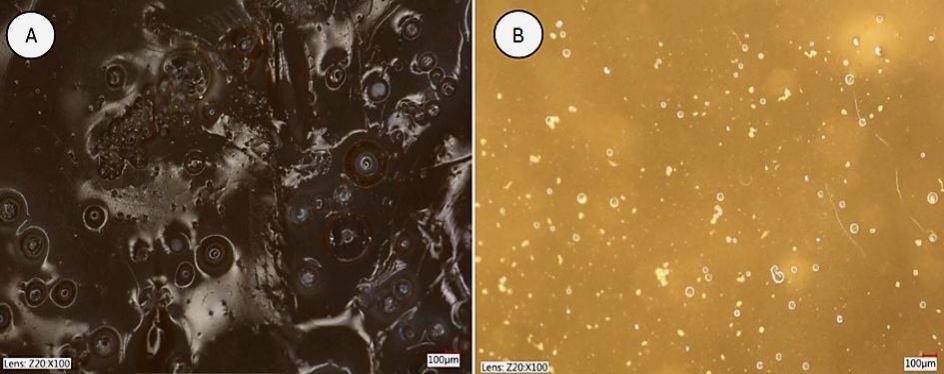
Microstructure of hydrogels at 100× magnification. A – alcohol-conditioned hydrogel; B – water-conditioned hydrogel

Numerous pores were identified in the horizontal cross-section of the cured sample, which made the hydrogel surface layer notably variegated. The horizontal cross-section of the reference sample showed few pores, which had a significant depth of about 300 μm despite their small diameter. The depth significantly exceeded the depth of the pores of the ethanol-immersed hydrogel sample, which occurred only on the surface. In the latter sample, local surface irregularity– related changes were shallower than those in the surface of the sample from the aqueous environment. The hydrogel immersed in alcohol also had a more compact and rigid structure, which was shown by the greater continuity of the surface visible under the microscope. Figure 9 compares the microstructure of hydrogels from different environments.

**Fig. 10.**
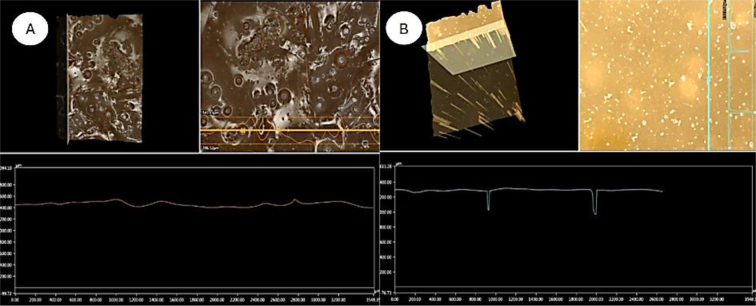
3D analysis proceeding from 100× magnification of pore distribution in hydrogel samples. A – alcohol-conditioned hydrogel; B – water-conditioned hydrogel

Samples conditioned in alcohol had significantly less closed porosity, resulting in a compact structure. More closed pores were observed in hydrogel samples conditioned in water. This is related to the vapour pressure, the evaporation temperature (which is lower for alcohol) and the lower vaporisation energy of alcohol compared to water. Figure 10 presents the measured roughness for the two types of hydrogel sample.

**Fig. 11.**
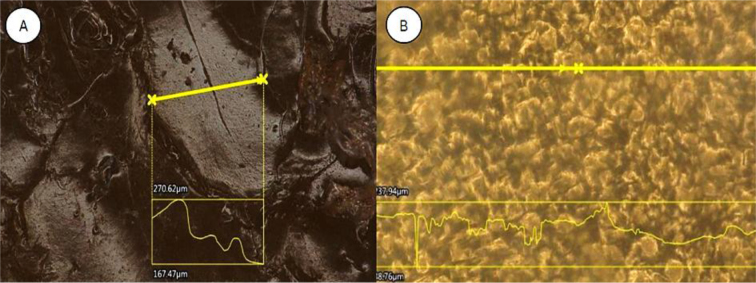
Microstructure of hydrogel samples at 200× magnification. A – alcohol-conditioned hydrogel; B – water-conditioned hydrogel

The roughness inspection showed that the sample conditioned in water contained open pores on its surface with a depth of 50–100 μm. These pores could be problematic in an implant of this type of hydrogel in the treatment of glaucoma, as they could harbour bacteria. The surface of the implant conditioned in alcohol was characterised by a closed and smoother (at most wavy) surface (Fig. 11).

**Fig. 12.**
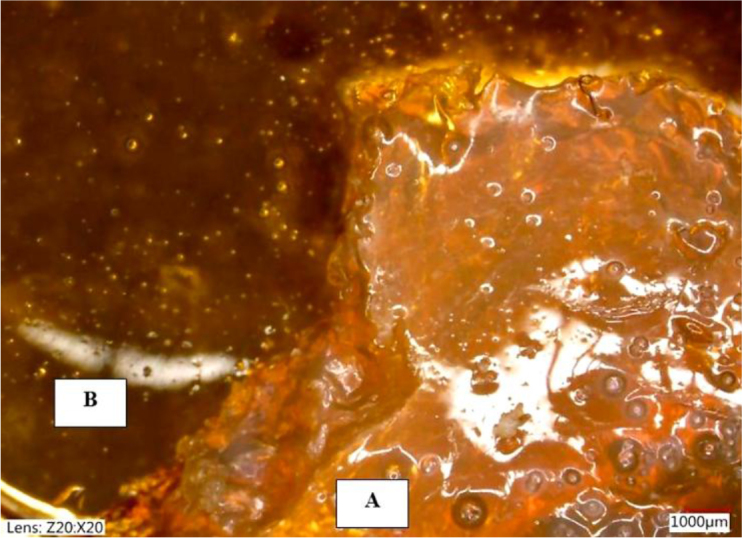
Comparison of microstructure of samples at 20× magnification A – alcohol-conditioned hydrogel; B – water-conditioned hydrogel

Figure 12 presents two types of hydrogel sample investigated for glaucoma treatment. It illustrates the general structural differences between the two hydrogel samples at lower magnification. The alcohol-conditioned sample displayed a more defined, opaque structure with visible surface irregularities, consistent with the denser, stiffer morphology resulting from alcohol-induced dehydration, while the water-conditioned sample appeared more transparent and structurally uniform, reflecting its higher water content.

## Discussion

The preliminary analysis of the shape retention and durability of the produced hydrogels immersed in water prompted us to base their formulation on chemically purified gelatine. As reported by Lv *et al*. (14), gelatine/polyacrylamide hydrogels with a double network achieved high compressive strength (up to 87 MPa) and high resistance, confirming that the condensing of the structure results in higher mechanical resistance. Analysis in the next stage revealed a positive effect of alcohol immersion of fresh hydrogels on their morphology and durability. The work of Boral *et al*. (2) on the osmotic behaviour of gelatine hydrogels cured in different concentrations of ethyl alcohol also showed that alcohol penetrated the structure and removed weakly bound water molecules, leading to pore shrinkage, which increased the density of the material. This was also confirmed by the studies of Wang *et al*. (23). Our static compression test showed that the hydrogel samples immersed in 99.8% ethyl alcohol had higher compressive strength than the samples of the same formulation immersed only in water. The alcohol environment caused the weakly bound water present in the hydrogel to pass into the alcohol and dissolve in it, which probably led to the closure of pores and thickening of gelatine chains, thus increasing the compressive strength. As reported by Karimi and Navidbakhsh (12), increased gelatine content increases Young’s modulus and compressive strength.

Reducing the free water content by dehydration stiffened the structure and reduced porosity, concordantly with the observed change in morphology after alcohol immersion noted by Gun’ko *et al*. (9). It was determined from the water’s evaporation energy that the amount in the hydrogel sample immersed in water was significantly greater than that in the hydrogel sample immersed in alcohol. This resulted from the effect of alcohol on the hydrogel structure. The surface of the sample immersed in ethyl alcohol was stiffer and less transparent than that of the sample immersed in water. Capillary pores of larger diameters were observed at higher microscopic magnifications on the surface of the latter hydrogel, which reduced the hydrogel’s mechanical strength. In contrast, smaller-diameter pores were seen on the surface of the alcohol-cured hydrogel as locally visible signs of closure of pores, which raised the hydrogel’s mechanical strength. This is also confirmed by the research of Manish *et al*. (16), which showed that a hydrogel with higher water content (approximately 90%) had a softer, hyperelastic structure, while hydrogels with 50–70% water were stiffer, demonstrating again that water reduction increases mechanical strength.

The study of ion permeation through hydrogel composites is crucial for understanding the potential for salt diffusion in an ocular implant. The faster the ion diffusion process and the shorter the time in which equilibrium is reached in the system, the better the hydrogel is. Considering that the efficiency of ion permeation through the hydrogel may decrease over time, it is important that a hydrogel achieves the highest efficiency at the very beginning. Measurement of the ionic conductivity of the alcohol-cured hydrogels formulated in their four marginally different ways showed that the obtained samples did not impede ionic transport in an aqueous environment. The research of Cui *et al*. (4) on gelatine-starch composites showed that the conditioning environment (e.g. ethanol) changes the surface microstructure and porosity, which affected ionic conductivity.

We conducted a detailed preclinical comparison of the developed implant with the XEN Gel Stent, the most commonly used commercial solution (Table 7). The XEN Gel Stent is a hydratable, flexible tube approximately 6 mm long with an internal diameter of 45 μm, made of porcine gelatine crosslinked with glutaraldehyde. In a clinical model, this stent reduces IOP through controlled fluid outflow at a rate calculated by the Hagen–Poiseuille equation, preventing excessive pressure drop (6–8 mmHg flow resistance). The material is characterised by high biocompatibility, minimal tissue reaction and high *in vivo* durability. Our implant was also manufactured from gelatine and glutaraldehyde, but in a tunnel-shaped design with a variable core diameter (approximately 1–3 mm), and was optimised for form and functionality, flexibility after hydration and potential economic benefits. The material allows adjustment of dimensions and composition, which provides advantages in fit and production costs. However, this implant requires further validation of its physicochemical properties, such as its actual Hagen– Poiseuille flow rate and hydrolytic stability, and the implant also needs confirmation of its cyto- and biocompatibility. In summary, the developed implant has promising translational potential because of its flexible design, locally available raw materials and cost-effective production. However, further *in vitro* and *in vivo* studies are necessary to confirm its efficacy, durability and safety compared to established solutions such as the XEN Gel Stent.

**Table 7.** Mechanical properties of the developed implant alongside those of available clinical solutions

	Designed implant	XEN Gel Stent	EX-PRESS	Baerveldt
Material	Gelatine + glutaraldehyde	Pork gelatine + glutaraldehyde	Stainless steel	Silicone
Inner dia	1–3 mm (core)	45 μm	50 μm	300 μm
Length	As needed	6 mm	2.64 mm	Various
Flexibility	High after hydration	10° (15 μN), 35° (70 μN)	Rigid	2,000 μN

The presented study has several significant limitations that impact the interpretation of the obtained results and their translation into clinical practice. First, the biological safety assessment did not include classic *in vitro* cytotoxicity tests (the 3-(4,5-dimethylthiazol-2-yl)-2,5-diphenyltetrazolium bromide (MTT), live/dead cell viability and direct contact assays called for by ISO 10993-5), nor did analysis take place of glutaraldehyde residues, which prevents us forming conclusions about the presence of potentially cytotoxic substances. Furthermore, the kinetics of hydrogel degradation under physiological conditions were not determined, making prediction of the stent’s long-term durability and degradation in the body impossible. Data on fluid flow through the implant derived from the Hagen–Poiseuille equation are also lacking, impeding the assessment of its potential as an IOP-regulating device. Regarding material preparation and characterisation, the inexhaustive surface analysis (microscopy only up to 200× magnification) did not allow the identification of fine structures (*e.g*. porosity) that may affect biocompatibility. There was also no validation of the sterilisation process or its effect on the physicochemical properties of the implant. No *in vivo* studies were conducted, leaving the actual biocompatibility, target species inflammatory and immunological responses or implant migration tendency unknown; however, assessing the body’s response to the material is a key step in the evaluation chain of ISO 10993-6 and other biocompatibility standards. Furthermore, the study does not include comparisons with commercially available devices, such as the XEN Gel Stent – there are no direct mechanical or flow tests, nor is there comparison of this stent’s production costs with those of technologies already implemented. Other significant limitations are the short observation time of the final candidate hydrogel (72 h) and the conduct of only a single run of the experimental stages, which weakens the statistical power of the results. There is also a lack of independent validation of the results by an external laboratory, which could confirm the repeatability and reliability of the obtained data. The remaining testing needs and the data shortfall necessitate extensive *in vitro* and *in vivo* studies, including investigations of the stent’s flow, cytotoxicity, immunocompatibility and degradation and finer characterisation of its morphology. No less necessary are a comparative analysis of the implant and existing commercial products and validation of the its manufacturing and sterilisation methodology. These undertakings will reliably assess the translational potential of this solution in veterinary glaucoma therapy.

## Conclusion

Composite hydrogels enable both mechanical reconstruction of the drainage structure and controlled, long-term release of drugs. Hydrogel glaucoma implants demonstrate high biocompatibility, structural stability and biodegradability along with effective lowering of intraocular pressure, which makes this direction very promising for veterinary glaucoma ophthalmology. In preclinical conditions, hydrogel implants appear to be a safe and minimally invasive solution supporting glaucoma therapy. For this reason, more research, improvement of the proposed implant and participation of animals in clinical trials are required, which could significantly accelerate the implementation of these innovations. The developed hydrogel material and the technology for producing tube implants made of it meet economic criteria and have promise for application in minimally invasive glaucoma surgery. The produced implant warrants further investigation, which could include the flow rate of the fluid and the means to downscale the device.
